# Protocol for assessing myogenic tone and perfusion pressure in isolated mouse kidneys

**DOI:** 10.1016/j.xpro.2024.102845

**Published:** 2024-01-30

**Authors:** Zhugang Chu, Mario Kassmann, Yoland-Marie Anistan, Friedrich C. Luft, Maik Gollasch, Dmitry Tsvetkov

**Affiliations:** 1 Department of Internal Medicine and Geriatrics, University Medicine Greifswald, 17489 Greifswald, Germany; 2 Department of Urology, Guizhou Provincial People’s Hospital, Guiyang 550000, China; 3 Experimental and Clinical Research Center (ECRC), a joint cooperation between the Charité Medical Faculty and the Max Delbrück Center for Molecular Medicine (MDC), Berlin, Germany

**Keywords:** Health Sciences, Model Organisms

## Abstract

The isolated perfused kidney is a classic *ex vivo* preparation for studying renal physiology in general and vascular function. Here, we present a protocol for assessing myogenic tone in isolated mouse kidneys as well as vasodilatory and vasoconstrictive responses, expressed as perfusion pressure. We describe steps for pre-operative preparation, kidney and renal artery isolation, and connection of renal artery with glass cannula. We then detail how to measure pressure changes in perfused kidneys and the myogenic tone.

For complete details on the use and execution of this protocol, please refer to Cui et al.^1^

## Highlights

• Accessing vascular smooth muscle function in a physiologically relevant context
• Recording of four kidneys in parallel for high-throughput data acquisition
• Detailed procedures from kidney isolation to data collection

## Abstract (editor highlights)

Publisher’s note: Undertaking any experimental protocol requires adherence to local institutional guidelines for laboratory safety and ethics.

## Graphical abstract

**Figure:**
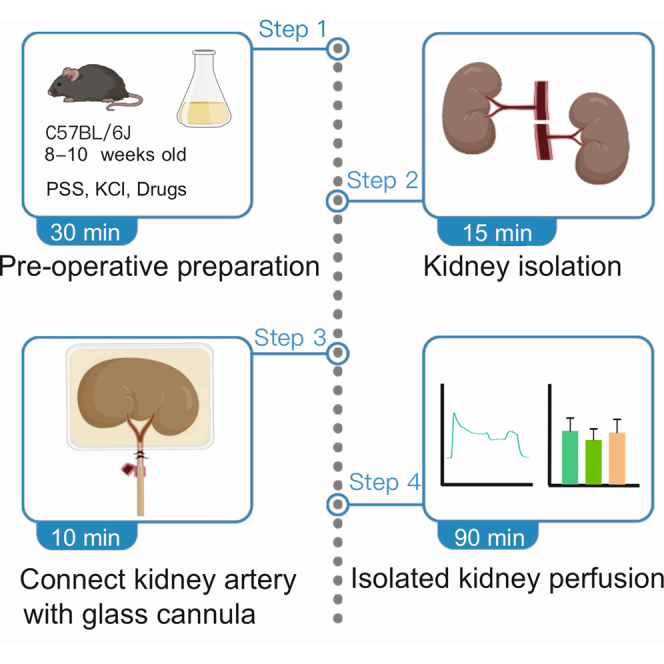

## Before you begin

Cardiovascular disease is the commonest cause of death worldwide^2^ and hypertension is the major driver.^3^ Since cardiac output remains normal in hypertension, systemic vascular resistance increases.^4^ To determine the underlying pathophysiology, small resistance vessels must be isolated and investigated. The isolated perfused kidney technique allows accessing vascular smooth muscle function in a physiologically relevant context and is superior to other *ex vivo* other methods, such as wire myography. After all, vessels act under pressure *in vivo* and are not stretched between wires.^5^ The isolated perfused kidney technique was first developed for use in large animals, but was successfully transferred to rats. The availability of gene-modified mice made murine models the ideal experimental animal model; however, substantial technical problems arose.^6^^,^^7^^,^^8^^,^^9^ Moving from 250 g rats (average kidney weight 2.5 g) to 25 g mice (average kidney weight 0.25 g) mice was a major challenge. Nonetheless, our approach is simple and allows the recording of four kidneys in parallel for high-throughput data acquisition. We describe our method including renal surgical preparation, artery isolation, and equipment assembly in detail (Graphical Abstract).

**CRITICAL:** To avoid renal artery damage, a comprehensive understanding of murine renal anatomy is essential.

***Note:*** Compared with the abdominal aorta and renal artery, the renal vein and inferior vena cava are larger, are located superficially, and have thinner walls. By removing the renal vein and underlying connective tissue, the location and shape of the renal artery and abdominal aorta can be clearly identified. The left renal artery typically lacks other major branches, whereas the right renal artery often has one or more branches. Identifying the branches of renal arteries to avoid their damage is essential.

### Institutional permissions

All animal care followed American Physiological Society guidelines, and all protocols were approved by local animal welfare officers and authorities (No. OE-017/23, LALLF: Landesamt für Landwirtschaft, Lebensmittelsicherheit und Fischerei Mecklenburg Vorpommern / State Office for Agriculture, Food Safety and Fisheries Mecklenburg-Western Pomerania), confirming that all experiments conform to the relevant regulatory standards. The described methods do not include experiments on live vertebrates or higher invertebrates. Depending on local regulatory rules, permissions from the relevant institutions might be required. There are no ethical concerns.

**Figure 1:**
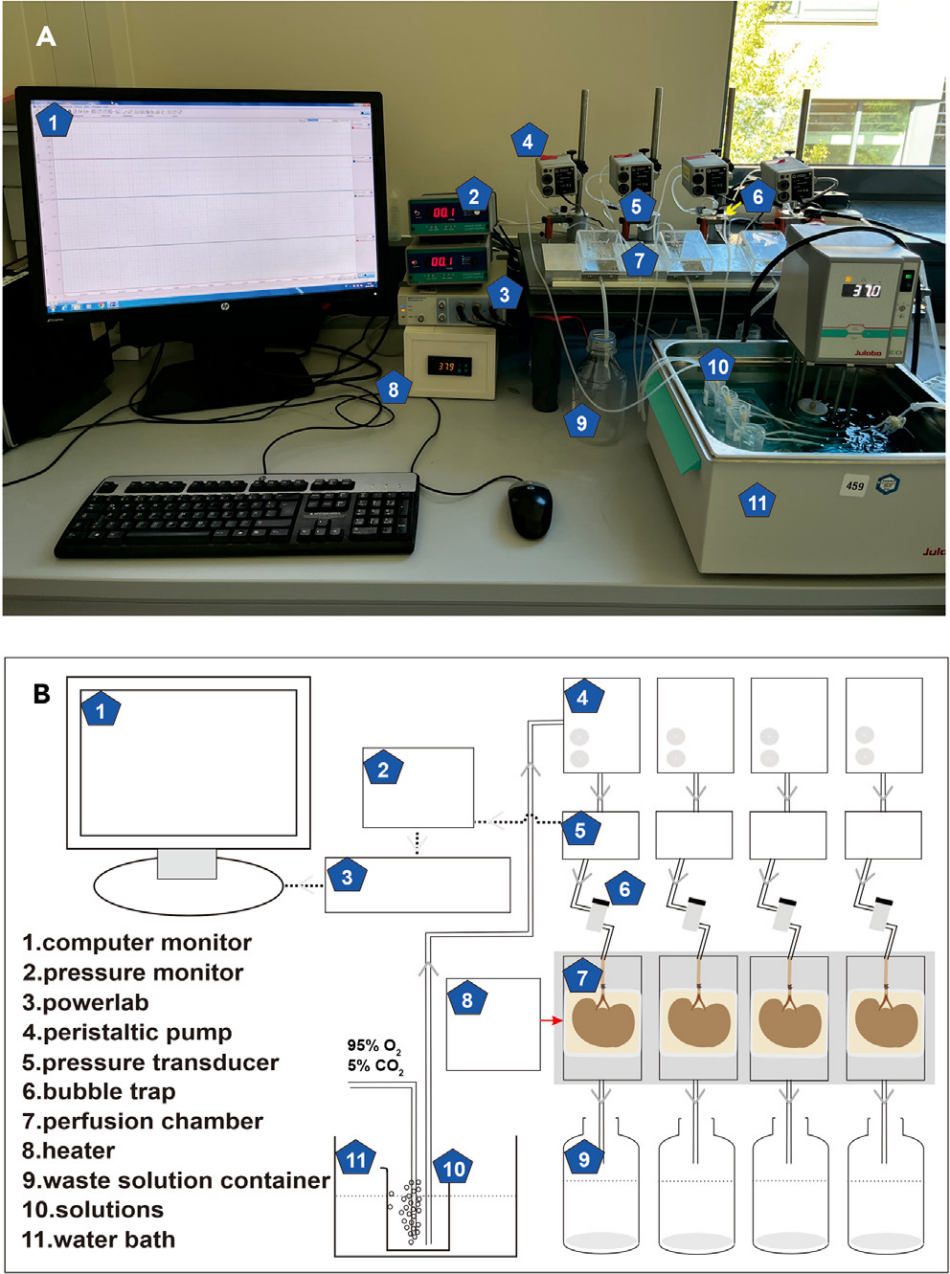
Isolated kidney perfusion setup (A) Assembled set up. (B) Schematic representation of the assembled unit.

1. Confirm the strain, sex, age, and weight of the mice.
2. Prepare the setup, and solutions needed according to Figure 1 and materials and equipment.

## Key resources table

**Table:** 

REAGENT or RESOURCE	SOURCE	IDENTIFIER
**Chemicals, peptides, and recombinant proteins**		

NaCl	Sigma-Aldrich	31434-1KG-R
NaHCO_3_	Sigma-Aldrich	S5761-5KG
KCl	Sigma-Aldrich	60130-1KG
MgSO_4_·7H_2_O	Merck	105886
D-Glucose	Sigma-Aldrich	G8270-1KG
CaCl_2_·2H_2_O	Roth	5239.1
KH_2_PO_4_	Roth	3904.1
Flupirtine	Sigma-Aldrich	F8927-5MG
Methoxamine	Sigma-Aldrich	M6524-500G
Ethanol (70%)	Carl Roth	200-578-6

**Experimental models: Organisms/strains**		

Mice, C57BL/6J strain. 8–12 weeks old, 25 g, female or male. Alternative strains can be used	The Jackson Laboratory	RRID:IMSR_JAX:000664

**Other**		

Surgical scissors	Fine Science Tools (FST)	15018-10
Forceps	FST	11254-20
Silk thread	RESORBA	6/0 USP, 0.7 metric
Silicon tubes	VWR International	228-5209 228-5208
PowerLab	ADInstruments	ML845 4/25, or PL2604 4/26 series, or PowerLab C or similar alternative data acquisition hardware can be used
Perfusion pressure monitor	Living Systems	PM-4
Glass cannula	Science Products	GB200-8P
Water bath	JULABO	12876
Micropipette puller	Narishige	PB-7 Vertical
Filter	Carl Roth	112A-240
Carbogene (95% O_2_/5% CO_2_)	Air Liquide	N/A
Peristaltic pump	Instech	Model P720
Replacement pressure transducer	Living Systems	PT-F
Bubble trap	Custom-made, alternatives can be used https://schmidt-haensch.com/product/glass-tube-with-bubble-trap/	N/A
Chamber	Custom-made, plexiglass, alternatives can be used	N/A
Chamber heater	Custom-made, alternatives can be used	N/A
LabChart	ADInstruments	v8.1.22, also lower and higher versions can be used

## Materials and equipment

**Table:** Physiological Salt Solution (PSS)

Reagent	Final concentration, mM	Amount
NaCl	119	13.91 g
KCl	4.7	0.71 g
KH_2_PO_4_	1.2	0.327 g
NaHCO_3_	25	4.2 g
MgSO_4_·7H_2_O	1.2	0.592 g
Glucose	11.1	4 g
CaCl_2_·2H_2_O	1.6	0.47 g
H_2_O	N/A	2000 mL
**Total**	**N/A**	**2000 mL**

Store at +4°C for up to 1 week.

**Table:** KCl 60 mM

Reagent	Final concentration, mM	Amount
NaCl	63.7	7.445 g
KCl	60	8.947 g
KH_2_PO_4_	1.2	0.327 g
NaHCO_3_	25	4.2 g
MgSO_4_·7H_2_O	1.2	0.592 g
Glucose	11.1	4 g
CaCl_2_·2H_2_O	1.6	0.47 g
ddH_2_O	N/A	2000 mL
**Total**	**N/A**	**2000 mL**

Store at +4°C for up to 1 week.

## Step-by-step method details

### Preparation of glass cannula

**Timing: 5 min**

**Figure 2:**
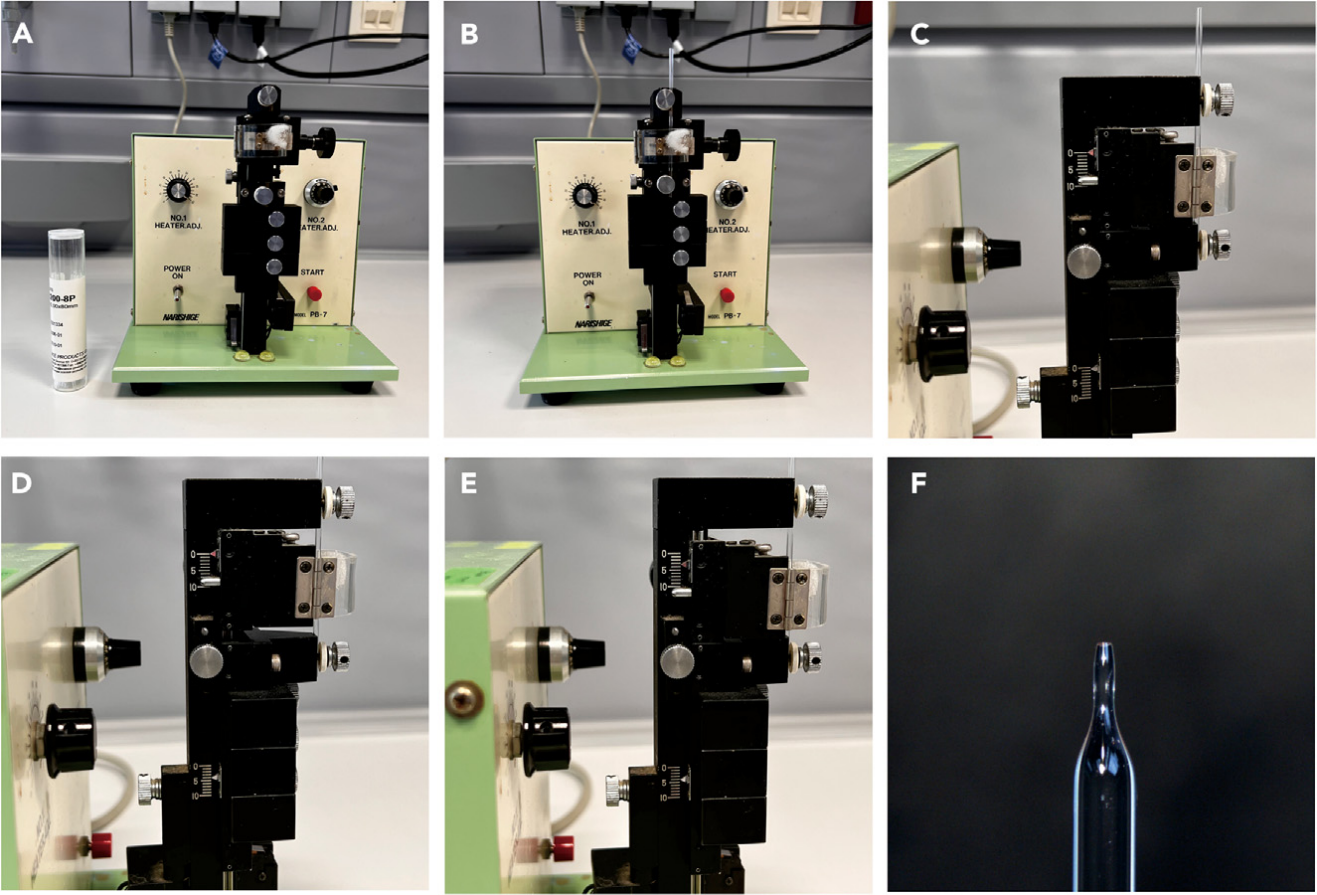
Process of glass cannula (A) Micropipette puller. (B) Glass is installed onto the puller. (C) Two scales of the puller are set to the starting position (0). (D) Heater is switched on and the lower scale is adjusted to 4 mm. After adjusting, the heater is switched off. (E) Heater is switched on and the upper scale is adjusted to 3.5 mm. After the cannula is made, the heater is switched off. (F) Thin part of the glass cannula is removed and the surface is polished.

This step describes the preparation of the glass cannula for renal perfusion.
  1. Prepare a glass cannula of the indicated shape using a micropipette puller (Figure 2A).
    a. Install the glass tube into the pulling head of the puller and make adjustments to ensure the proper positioning of the glass material (Figure 2B).
    b. Set both scales of the puller to the starting position (0). This step allows finer adjustments of the distance during the pulling process that defines the shape of the cannula (Figure 2C).
    c. Turn on the heater and allow the lower scale descend for 4 mm. Fix the lower part of the tube holder and then turn the heater off (Figure 2D).
    d. Adjust the upper scale of the tube holder to 3.5 mm (Figure 2E). Switch on the heater again and gradually pull the glass material until the glass tube is separated into 2 parts.
    e. After completing of the pulling process, remove the longer cannula from the puller. Trim any excess of the tube and polish the surface of the cannula using a smooth metal surface (e.g., forceps) (Figure 2F).
    f. Verify the inner diameter of the tube according to the experimental requirements.

**CRITICAL:** The inner diameter of the glass cannula tip is crucial. A larger cannula diameter can make the connection with the renal artery unreliable, whereas a smaller diameter might significantly increase the resistance. The optimal inner diameter of glass cannula for mouse isolated kidney perfusion should range between 0.3 mm to 0.4 mm.

### Pre-operative preparation

**Timing: 30 min**

This section describes preoperative preparation procedures.

2. Oxygenate solutions.
  a. Set the water bath temperature to 37°C.
  b. Place solutions including physiological salt solution PSS and 60 mM KCl in the water bath.
  c. Place carbogen (95% O_2_ and 5% CO_2_) tubes into the solution bottles.
  d. Place a small bottle of PSS solution on ice and connect it to carbogen.

***Note:*** The solutions need to be oxygenated for at least 15 min before the kidney isolation.

3. Prepare required equipment.
  a. Turn on the temperature control device of the chambers and set it to 37°C (Figures 1A and 1B: module 8).
  b. Switch on the pressure monitor (Figures 1A and 1B: module 2) and peristaltic pump (Figures 1A and 1B: module 4).
  c. The solution must fill the entire tubing system. Then, pause the peristaltic pump and set pressure monitor to 0 mm Hg.
4. Open the LabChart software, set the pressure measurement range, and parameters such as the number of chambers (4 in our case).

### Kidney isolation

**Timing: 5 min**

In next two sections, we describe surgical techniques related to kidney isolation and isolation of renal arteries (Methods video S1).

**Figure 3:**
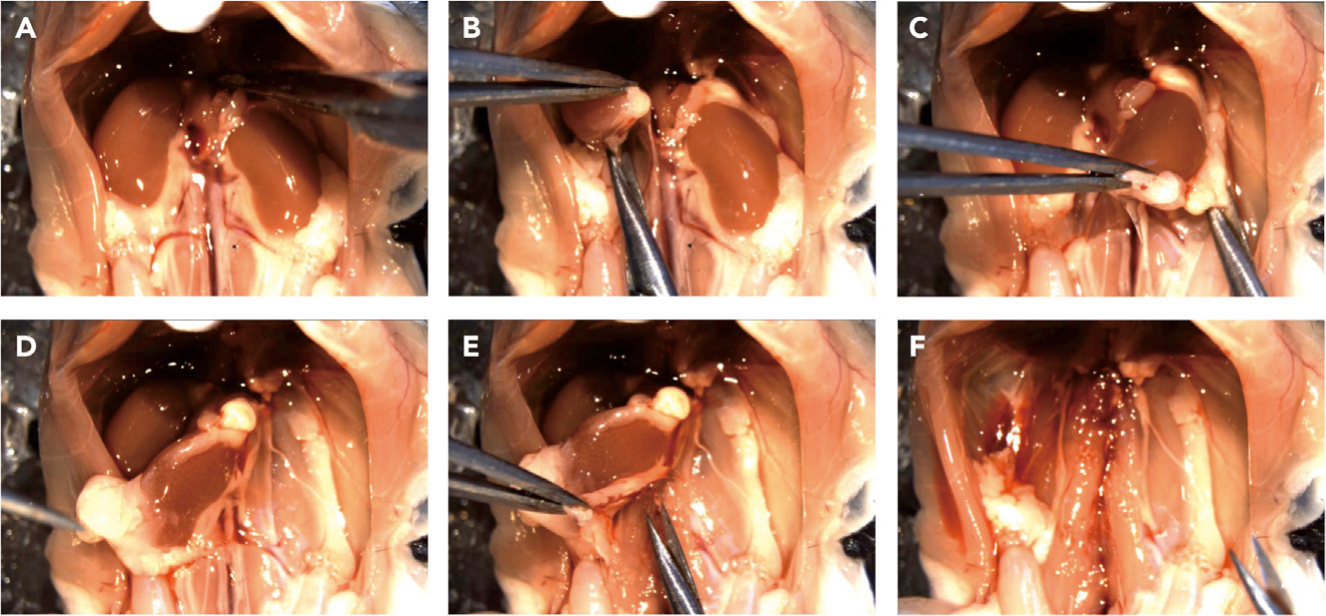
Kidney isolation (A) Cutting the aorta and vena cava inferior, after the stomach and intestine were removed or moved to the side. (B) Separation of the right and left kidney (C) from underlying connective tissue. (D) Flipping the left kidney onto the right side. (E) Separation of kidneys, abdominal aorta, renal artery, and inferior vena cava from connective tissue and fascia. (F) Kidneys are removed.
  c. Using forceps, take the lower part of right kidney of the mouse and lift it up. Using scissors, dissect the connective tissue on the dorsal side of the right kidney from bottom to top (Figure 3B).
  d. Repeat the same procedure with the left kidney (Figure 3C) and move it to the right side (Figure 3D).
  e. Once the aorta is exposed, grab the abdominal aorta and inferior vena cava at the level of the lower kidney part and cut them.
  f. Next, delicately separate them from the underlying muscle and connective tissue (Figure 3E).
  g. Once the kidneys are removed (Figure 3F), place them into a plate filled with cold oxygenated PSS solution (see step 1, preoperative preparation).

5. Isolate mouse kidneys.
  a. Open the abdominal cavity of a sacrificed mouse to expose internal organs such as intestine and liver. Gently displace the intestine and part of the liver towards the left side to visualize both kidneys.
  b. Carefully transect the connective tissue, aorta, vena cava inferior above the kidneys. (Figure 3A).

**CRITICAL:** During this procedure, there is a risk of damaging renal arteries. A useful technique is to lift the abdominal aorta while dissecting, and to cut as close as possible to the underlying muscle (Methods video S1, 1 min 10–40 s).

Methods video S1. Kidney isolation, related to step 5

### Isolation of renal arteries

**Timing: 10 min**

**Figure 4:**
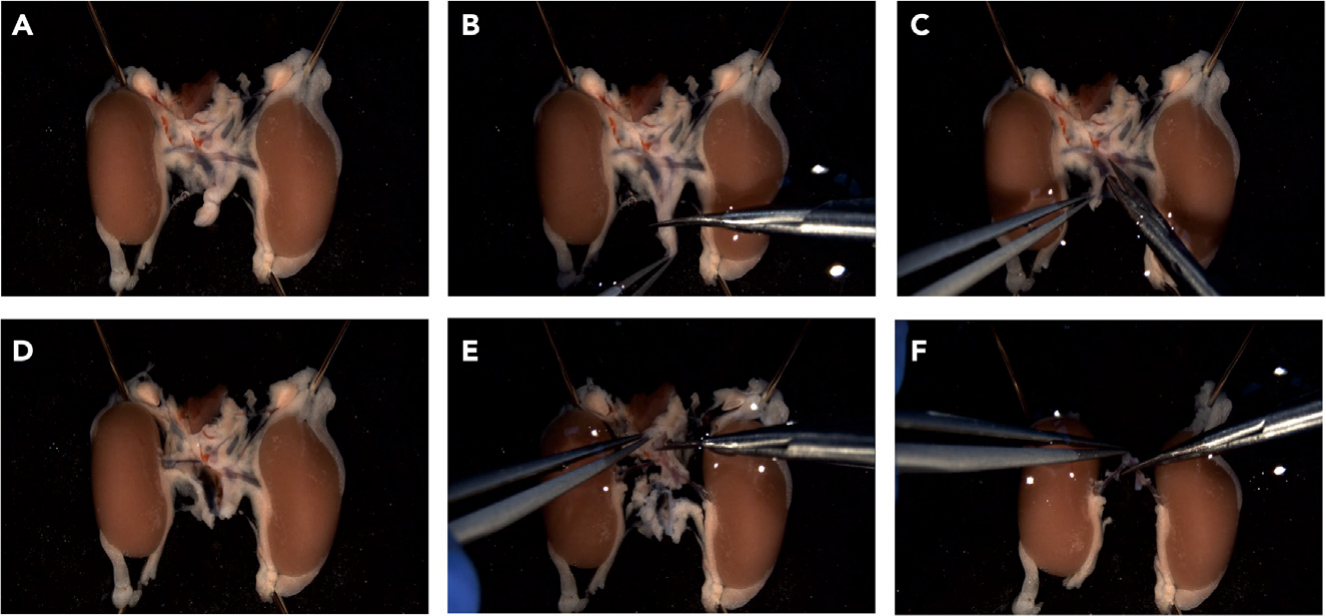
Isolation of renal arteries (A) Fixation of the kidneys with needles onto a custom-made Sylgard plate. (B) Cutting off the lower part of the aorta, inferior vena cava and surrounding tissue with scissors. (C) Incision of the inferior vena cava and (D) subsequent right renal vein with removal of surrounding connective and perivascular adipose tissue. (E) Removing excess of abdominal aorta with connective tissue; the part of aorta with both renal arteries is left intact. (F) All connective and perivascular adipose tissue are removed; the two renal arteries are separated from the aorta.
  b. Remove an excess of abdominal aorta (Figure 4B).
  c. Make an upward incision along the vena cava inferior towards 11 o’clock (Figure 4C).
  d. Extend this incision to the right renal vein using scissors to clearly visualize the right renal artery (Figure 4D). Apply same procedure to left kidney vein.
    **CRITICAL:** The renal vein is semitransparent, aim to make incisions within this translucent area to avoid damaging the branches of underlying renal artery.
  e. Use scissors to remove surrounding connective and adipose tissue of renal arteries and aorta (Figure 4E).
  f. Separate two renal arteries from the aorta (Figure 4F).
  Methods video S2. Isolation of renal arteries, related to step 6

6. Isolate renal arteries (Methods video S2).
  a. Use needles to fix adipose tissue at the upper and lower margins of the kidneys, maintaining slight tension on the renal artery (Figure 4A).

### Connection of renal artery with glass cannula

**Timing: 5 min**

This section describes how to connect the mouse renal artery to the glass cannula and the pressure-monitoring device (Methods video S3).

**Figure 5:**
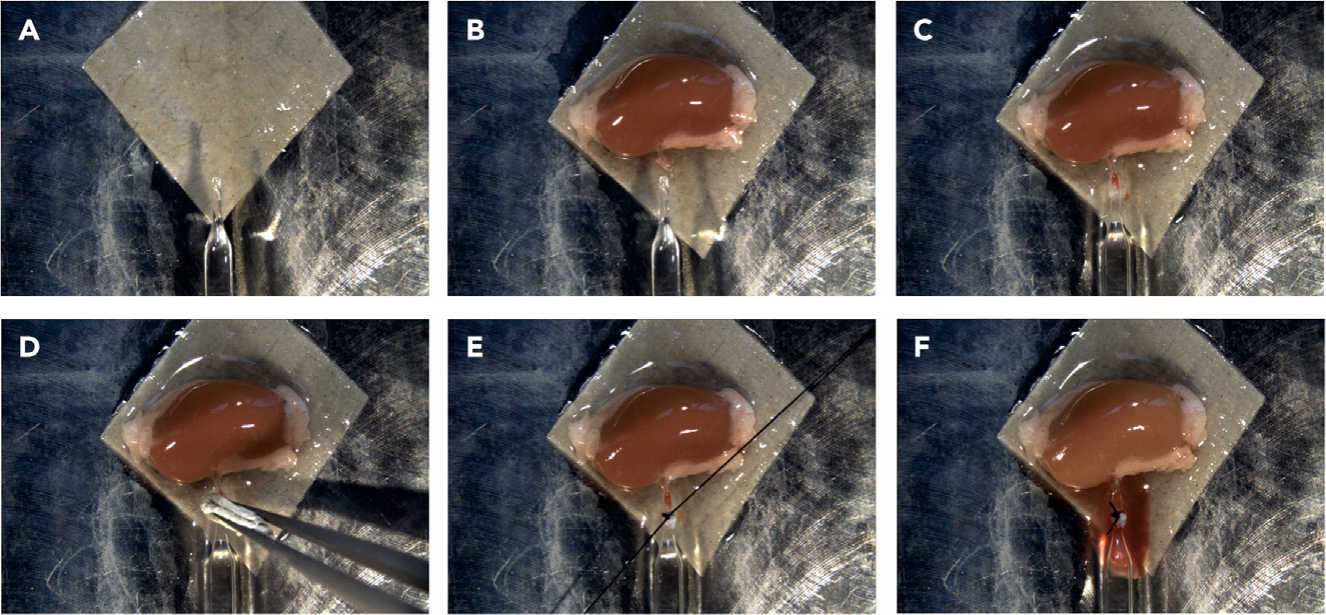
Connection of renal artery with glass cannula (A) Purging air from the glass cannula with PSS through a syringe; a part of a paper towel is placed on the platform. (B) Placing the kidney on the platform. (C) Connecting the renal artery with the glass cannula. (D) Using a part of a paper towel to position the renal artery appropriately. (E) Securing the renal artery with a surgical knot. (F) Condition of the kidney after perfusion with PSS through a syringe and removing the excess of silk material.
  b. Place the kidney in the chamber on paper tissue, ensuring that the renal artery is oriented towards the glass cannula (Figure 5B). Either left or right kidneys can be used.
  c. After adjustment of the position and the angle of the kidney by moving the paper tissue, use forceps to place renal artery on the glass cannula (Figure 5C).
  d. Use a piece of paper and forceps to adjust the position the renal artery (Figure 5D) (Methods video S3: 25–35 s).
    **CRITICAL:** During this procedure, to avoid grasping the artery with the forceps is crucial, since the renal artery can be damaged.
  e. Move the knot to fix the renal artery to glass cannula, and securely tie it (Figure 5E).
  f. Gently inject cold oxygenated PSS solution into the glass cannula using a syringe. Successful cannula connection to the renal artery and the kidney is indicated by a pale color of the whole kidney and lack of leakage (Figure 5F).
    ***Note:*** If only part of the kidney appears pale after PSS infusion, the result could indicate that the glass cannula is located in a branch of the renal artery. Adjusting the catheter's position might address this problem.

7. Connect renal arteries with glass cannula.
  a. Secure the glass cannula in the chamber. Make a knot using silk thread and place it over the cannula for future use. Rinse the cannula with PSS to remove air bubbles from the cannula (Figure 5A).

Methods video S3. Connection of renal artery with glass cannula, related to step 7

### Pressure changes in isolated perfused kidneys

**Timing: 2 h**

In this section, we describe the protocol of measuring the perfusion pressure in isolated kidneys due to pharmacological activation of KCNQ channels (K_V_7 family of voltage-gated potassium channels). The background information is provided in the expected outcomes section.

8. Measure the pressure changes in isolated perfused kidneys.
  a. Using peristaltic pump, perfuse the kidney with very low flow rate (0.3 mL/min) for 45 min - 1 h.
  b. Set the flow rate of peristaltic pump to achieve the stable perfusion pressure of 80 mm Hg (∼15 min)
  c. Perfuse the kidney with 3 μM Methoxamine (ME, a1-adrenoreceptor agonist) solution until the pressure reaches the plateau.
    ***Note:*** It is recommended to wait until stable pressure - plateau is reached (ca. 5 min).
  d. Perfuse the kidney with 3 μM ME and drug of interest (e.g., KCNQ opener, 10 μM Flupirtine)
  e. Wash out the KCNQ opener by perfusing the kidney with 3 μM ME
  f. Wash out the ME by perfusing the kidney with PSS
  g. Perfuse the kidney with 60 mM KCl.

### Myogenic tone

**Timing: 2 h**

In this section, we describe the protocol of measuring the myogenic tone. The background information is provided in the expected outcomes section.

9. Measure the myogenic tone.
  a. Perfuse the kidney with very low flow rate (0.3 mL/min) for 45 min - 1 h.
  b. Stepwise increase the flow rate (0.7; 1.3; 1.9 mL/ min). The duration of each step can vary and depends on reaching the plateau (ca. 3–5 min).
  c. Reduce the flow rate to 0.5 mL/min until the pressure reaches a plateau (∼10 min).
  d. Perfuse the kidney with 10 nM Ang II until the pressure reaches a plateau (∼5 min)
  e. Wash out the Ang II by perfusing the kidney with PSS.
  f. Perfuse the kidney with Ca^2+^ free PSS until the pressure reaches a plateau (∼10 min).
  g. Wash out Ca^2+^ free PSS with PSS until the pressure reaches a plateau (∼10 min).
  h. Perfuse the kidney with 60 mM KCl until the pressure reaches a plateau (∼5 min).

## Expected outcomes

### Pressure changes in isolated perfused kidneys

Small-artery and resistance-vessel regulation is the key element regulating blood pressure and blood flow.^10^^,^^11^ Systemic vascular resistance in hypertension “is not everything; it is the only thing”. Vasoconstriction is mediated by increased intracellular Ca^2+^ concentration in vascular smooth muscle cells (VSMCs). This result is achieved by promoting the entry of extracellular calcium ions into the cells and releasing calcium ions stored within the cells.^10^^,^^12^^,^^13^^,^^14^^,^^15^^,^^16^^,^^17^

**Figure 6:**
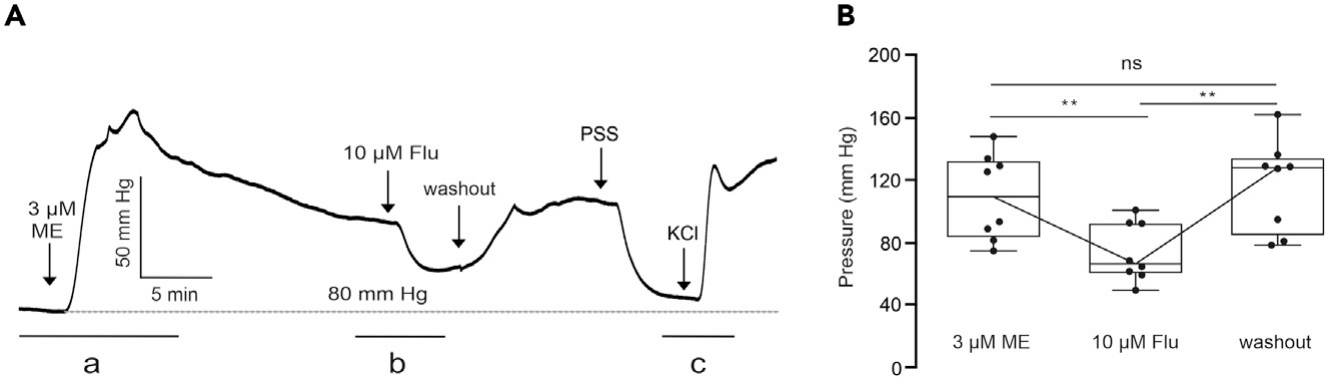
Expected results: impact of methoxamine (ME), flupirtine (Flu), and KCl on renal perfusion pressure (A) Original recordings of the perfusion pressure in kidneys using 3 μM ME, 10 μM Flu, subsequent washout, and 60 mM KCl. (B) Changes in the perfusion pressure induced by ME (increase), Flu (decrease) and subsequent washout procedure (increase). ∗∗P < 0.01; n.s., not significant; PSS, physiological saline solution.

Once the perfusion pressure has stabilized at the level of 80 mm Hg, 3 μM Methoxamine (ME) is added to perfusate (Figure 6, part a). Methoxamine binds α1-adrenergic receptors in vascular smooth muscle cells, activating Gq signaling pathway. Although the detailed mechanism is a matter of debate, this effect is expected to cause vasoconstriction and increase the perfusion pressure (Figure 6, part a).^18^ After reaching a plateau, 10 μM Flupirtine (KCNQ channel agonist) is added to perfusate. This result is expected to increase open probability of KCNQ channels and enhance efflux of potassium ions (K^+^) from the cells causing VSMCs hyperpolarization. The hyperpolarized cell membrane closes VGCCs, reducing intracellular calcium ions (Ca^2+^) concentration. Therefore, it is expected to cause vasodilation and a decrease in peripheral vascular resistance, reducing the perfusion pressure. (Figure 6, part b). If the binding to KCNQ channel is reversible, after Flupirtine is washed out perfusion pressure should increase again. Finally, 60 mM KCl is added to perfusate (Figure 6, part c).

High extracellular K^+^ concentration results in membrane depolarization and an increased opening probability of VGCCs. Intracellular calcium ion (Ca^2+^) level rises, which is followed by smooth muscle contraction. As consequence, vasoconstriction and an increase in peripheral vascular resistance occurs, thereby the increasing of perfusion pressure is expected. This step helps to ensure that there is no leakage between glass cannula and renal artery and the VSMCs are fully functional. The last steps (5–7) in the protocol 1 are optional. Difference in pressure can be calculated and represented as shown (Figure 6B).

### Myogenic tone

**Figure 7:**
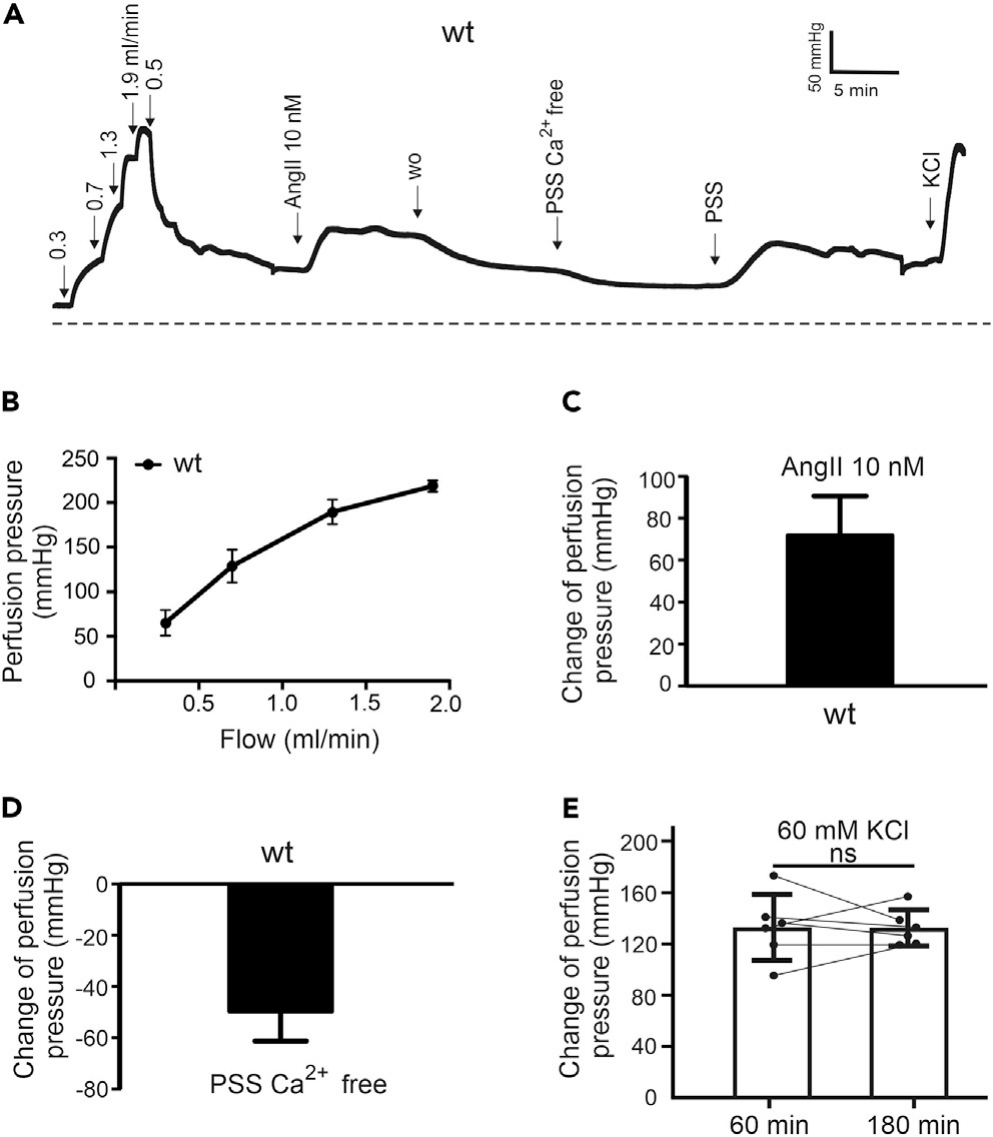
Myogenic tone (A-C) Original recordings of the perfusion pressure in kidneys (A). Increase in perfusion pressure induced by increased flow rate (B) and 10 nmol/L Ang II (C). (B and C) Data are mean ± SEM. (D) Change of pressure in response to Ca^2+^ free PSS. Data are mean ± SEM. (E) Increase in perfusion pressure induced by 60 mM KCl at 60 min and 180 min n = 6, kidneys from N = 6 mice, n.s., not significant as determined by paired t-test. Error bars represent 95% confidence interval (CI) of mean.

Elevation of intravascular pressure causes constriction (myogenic tone) of small arteries and arterioles. The smooth muscle of both large arteries and small arterioles constrict in response to increased pressure and dilate in response to decreased pressure, a phenomenon known as the “Bayliss effect”.^19^ This effect and has been observed in various microvascular beds.^20^ In the initial stage, the kidney is perfused at a relatively low-flow rate to achieve equilibration. Subsequently, the flow rate is gradually increased (0.3; 0.7; 1.3; 1.9), leading to an increase in the amount of fluid passing through the blood vessels per unit time (Figures 7A and 7B).^1^ The flow rate is then adjusted to 0.5 mL/min, causing the perfusion pressure to decrease. Next, angiotensin II (Ang II) is applied. The peptide binds to angiotensin II type 1 receptor, which activates Gq/11-dependent signaling pathway, thereby increasing intracellular Ca^2+^ concentration in vascular smooth muscle cells *via* the entry of extracellular calcium ions into the cells and the release of calcium ions stored within the cells. The increased calcium concentration stimulates the binding of actin and myosin, leading to subsequent vasoconstriction, resulting in an elevation of perfusion pressure. When changing to Ca^2+^ free PSS, no extracellular Ca^2+^ ions are available, which explains the decrease in perfusion pressure. This difference gives a measure of myogenic tone and helps to differentiate these state-of-the-affairs from passive dilation. Difference in pressure can be calculated and represented (Figures 7C and 7D).

## Limitations

In summary with our protocol, whole kidneys can be studied in a (murine) rodent vascular model. Our interests are directed at small resistance vessels and not at basic renal physiology. Neither venous collection, nor urine collection are addressed using this technique, although these should be possible. Potential limitations of isolated kidney perfusion experiments include the prolonged use of colloid-free and cell-free solutions. Repetitive use of PSS may result in tissue edema, affecting hemodynamics, and renal function. These confounding variables could influence the accuracy of perfusion pressure measurements. Therefore, to ensure the reliability and accuracy of the results, awareness and control of these potential limitations is crucial. Fresh solutions should be prepared. Moreover, the composition of the perfusion fluid (PSS) may potentially influence the physiological state and vascular reactivity of the kidneys. Using PSS as a substitute for whole blood may not fully replicate *in vivo* physiology, leading to inaccuracies in assessing vascular responsiveness.^21^ In isolated rat kidney perfusion, removing the renal capsule during preparation may reduce the impact of tissue edema.^22^ Adding a erythrocyte concentrate or albumin solution could help to overcome these issues.^23^ However according to our experience, acute experiments of 3 h duration did not cause substantial problems regarding the vascular resistance, since the vascular response to 60 mM KCl remained unchanged (Figure 7F).

## Troubleshooting

### Problem 1

Fluctuations in perfusion pressure (increase).

### Potential solution

• Due to small size of capillaries within the kidney, dust particles in the perfusion solution can block the capillaries. This effect increases perfusion pressure. Before the experiment, perfusion solutions should be filtered using filter-paper. To avoid possible binding of drugs to filter paper and to ensure the correct drug concentrations, we suggest filtering PSS solutions and using the filtrate for further drug dilutions.
• In addition to dust, air bubbles may increase pressure. In this case, we recommend adjusting the solution level in the bubble trap (Figure 1).

### Problem 2

Fluctuations in perfusion pressure (decrease).

### Potential solution

• Most often, a leakage causes the pressure instability. Familiarize yourself with the anatomy of the mouse blood vessels. Avoid damaging the arteries when dissecting the surrounding adipose and connective tissues. Often, it is possible to position the knot in order to close the leakage in the corresponding small branch of the renal artery.

## Resource availability

### Lead contact

Further information and requests for resources and reagents should be directed to and will be fulfilled by the lead contact, Dmitry Tsvetkov (dmitry.tsvetkov@med.uni-greifswald.de).

### Technical contact

Technical questions on executing this protocol should be directed to and will be answered by the technical contact, Dmitry Tsvetkov (dmitry.tsvetkov@med.uni-greifswald.de).

### Materials availability

This study did not generate new unique reagents.

### Data and code availability

Additional data are available are available from the lead contact upon reasonable request. This study did not generate new codes.
